# Acute Movement Disorders in Childhood

**DOI:** 10.3390/jcm10122671

**Published:** 2021-06-17

**Authors:** Giacomo Garone, Federica Graziola, Melissa Grasso, Alessandro Capuano

**Affiliations:** 1 Movement Disorders Clinic, Department of Neurosciences, Bambino Gesù Children’s Hospital, IRCCS, viale San Paolo 15, 00146 Rome, Italy; giacomo.garone@opbg.net (G.G.); federica.graziola@opbg.net (F.G.); melissa.grasso@opbg.net (M.G.); 2 University Department of Pediatrics, Bambino Gesù Children’s Hospital, 00165 Rome, Italy

**Keywords:** movement disorders, acute, emergency, children, autoimmune, chorea, dystonia, parkinsonism, drug-induced

## Abstract

Acute-onset movement disorders (MDs) are an increasingly recognized neurological emergency in both adults and children. The spectrum of possible causes is wide, and diagnostic work-up is challenging. In their acute presentation, MDs may represent the prominent symptom or an important diagnostic clue in a broader constellation of neurological and extraneurological signs. The diagnostic approach relies on the definition of the overall clinical syndrome and on the recognition of the prominent MD phenomenology. The recognition of the underlying disorder is crucial since many causes are treatable. In this review, we summarize common and uncommon causes of acute-onset movement disorders, focusing on clinical presentation and appropriate diagnostic investigations. Both acquired (immune-mediated, infectious, vascular, toxic, metabolic) and genetic disorders causing acute MDs are reviewed, in order to provide a useful clinician’s guide to this expanding field of pediatric neurology.

## 1. Introduction

Acute-onset movement disorders (MDs) are an increasingly recognized neurological emergency in both adults and children [1,2]. Prompt recognition and appropriate management of acute-onset MDs is crucial, particularly for treatable ones. Nevertheless, the literature about MD emergencies in children and adolescents is scattered. Few cohort studies are available, diverging in terms of recruiting setting, inclusion criteria and sample size [3,4,5]. Despite the lack of robust epidemiologic data, acute-onset hyperkinetic MDs have been reported to account for 0.6% of pediatric emergency consultations in one study [5]. No data are available for hypokinetic disorders, the rarest of pediatric MDs.

Given the vulnerability of the basal ganglia to different *noxae*, a broad range of neurological and systemic diseases may result in acute-onset MDs. Three different clinical scenarios are possible: (1) the acute or subacute appearance of a novel-onset MD, (2) the acute or subacute exacerbation of a chronic, pre-existing MD; and (3) the occurrence of a paroxysmal MD—a disturbance that has a discrete timing of onset and cessation. In this latter case, patients frequently come to medical attention after the resolution of symptoms.

In this review, we summarize the most common causes of acute, novel-onset MDs in children, focusing on clinical presentation and appropriate diagnostic investigations. Paroxysmal MD and *status dystonicus* are not treated, as they have been extensively reviewed elsewhere [6,7,8,9].

## 2. Methods

A bibliographic search on PubMed was performed on 1 May 2021 using key terms related to our review. No temporal filter was applied, but only English articles were considered. We searched for the terms “acute-onset”, “movement disorders”, “children”, “adolescents”, “dystonia”, “chorea”, “myoclonus”, “tics”, “parkinsonism”, “drug-induced”, “autoimmune”, “Sydenham”, “encephalopathy”, “metabolic”, “infections”, “encephalitis”, “meningitis”, “functional”, “stroke”, “Moyamoya”, both individually and in combination.

Both articles (research articles, reviews, case series or case reports) and book chapters were included in the final reference list.

## 3. Approach to Acute Movement Disorders in Childhood

**Figure 1:**
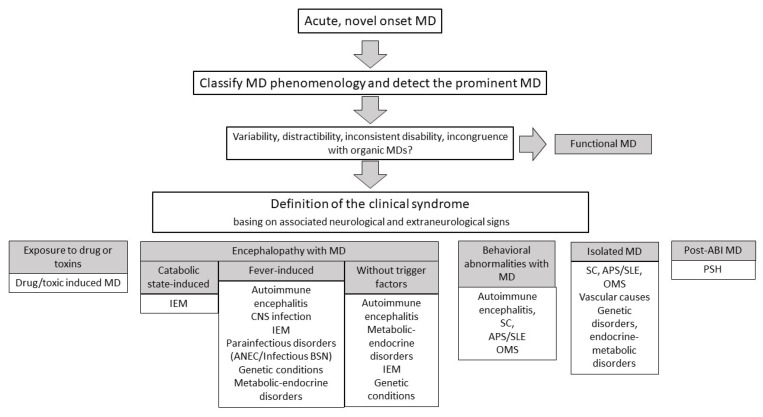
Clinical approach to acute-onset movement disorders. Based on the frequent clinical scenarios, the most relevant differential diagnoses are indicated. ANEC: acute necrotizing encephalopathy; APS: antiphospholipid syndrome; BSN: bilateral striatal necrosis; CNS: central nervous system; IEM: inborn errors of metabolism; OMS: opsoclonus–myoclonus syndrome; PSH: paroxysmal sympathetic hyperactivity; SC: Sydenham chorea; SLE: systemic lupus erythematosus.

The acute appearance of an MD is a challenging clinical scenario. The range of possible etiologies is wide, and a conspicuous proportion of the cases are explained by individually rare disorders [3,4,5]. As further detailed below, the same disease may present with different MDs, and the same clinical scenario may underlie different conditions. In addition, the a priori probability of a given diagnosis greatly changes according with age. As a result, no diagnostic algorithm may be applied to acute-onset MDs from birth to adolescence. Nevertheless, as previously described for chronic MDs [10], some general rules can be useful to build a rigorous but practical approach and can be applied with some differences to acute-onset MDs (Figure 1) [10]. The definition of the prominent MD phenomenology in the setting of a specific clinical syndrome is the paradigm according to which further investigations (if necessary) are considered, always prioritizing potentially treatable causes.

In some cases, the clinical scenario is highly suggestive of a specific diagnosis (e.g., focal dystonia rapidly emerging after neuroleptics assumption, or acute-onset chorea appearing a few weeks after a streptococcal pharyngitis), making further investigations unnecessary or easily tailored to the diagnostic hypothesis (see the text and Supplementary Table S1). Similarly, functional MDs can be positively recognized according with specific clinical features (see below), and unnecessary investigations to exclude organic causes should be avoided.

As a rule, neuroimaging is necessary in all other cases—especially when facing unilateral MDs—to exclude structural lesions. Routine blood tests including full blood count, glucose and electrolytes levels, blood gas, liver and kidney function tests should be always performed to detect metabolic derangements and may provide elements to suspect an inborn error of metabolism (IEM). In the case of impaired consciousness, an EEG may prove extremely helpful to assess the severity of the acute encephalopathy, to detect unrecognized epileptic activity and to identify EEG patterns orientating towards a specific diagnosis [11]. In the case of fever-induced encephalopathy with MDs, cerebrospinal fluid (CSF) sampling should never be delayed, and the exclusion of infectious causes must be prioritized. If clinical picture, EEG and/or CSF findings point toward an encephalitic process, but no definite microbiological diagnosis can be reached, oligoclonal bands and antibody testing for autoimmune encephalitis should be always performed. For this eventuality, it may be useful to stock a small amount of CSF for further investigations after every lumbar puncture for suspected encephalitis. In young children with acute encephalopathy induced by catabolic states (fever, infections, fasting) or high protein intake, screening for IEM on blood, urine and CSF should be always performed. Table 1 catalogues a non-exhaustive list of useful investigations in acute-onset MDs.

## 4. Immune-Mediated Movement Disorders

### 4.1. Sydenham Chorea

Sydenham chorea (SC) is the most common cause of acute chorea in children [12] and one of the most common acute MDs overall (illustrative case 1, Supplementary Material) [3,4,5]. As one of the cardinal manifestations of rheumatic fever (RF), it develops usually 4–8 weeks after a Group A β-Hemolytic streptococcal (GABHS) infection [13]. The age of onset usually ranges between 5 and 14 years, with a peak at 8–9 years [14]. Although possible, onset under 5 years [13,14] or in young adulthood [15] (where relapses of pre-existing SC are more common) are quite rare [15]. Females are more affected than males (3:1 ratio) [12]. Usually, choreic movements rapidly evolve into a generalized chorea, although 20% of patients have unilateral or strongly asymmetrical involvement [16]. Other motor symptoms include motor impersistence, dysarthria, impaired saccades, tics, oculogyric crisis and hypotonia, that in rare cases may be so severe to cause a flaccid quadriplegia (*chorea paralytica*) [15]. Non-motor symptoms may precede the onset of chorea and encompass obsessive-compulsive disorders, attention deficit and hyperactivity, anxiety, emotional lability, depression and dysexecutive functioning [17,18].

The diagnosis of SC relies on the demonstration of Jones’ criteria for RF [19], in the absence of an alternative cause for chorea. Carditis and arthritis are associated in 60–80% and 20% of the cases, respectively [15]. Diagnostic work-up includes the demonstration of previous GABHS infection (through throat swab culture and determination of anti-streptolysin O and anti-DNAse B titers), cardiac evaluation for rheumatic carditis (including electrocardiogram and echocardiogram) and assessment of inflammatory markers (C-reactive protein and erythrocyte sedimentation rate). However, due to the long latency, evidence of the inciting GABHS infection may be difficult to obtain at SC onset. Cerebrospinal fluid (CSF) analysis and neuroimaging do not usually provide diagnostic or prognostic information, but they may help in rule out alternative causes [15].

Beside antibiotic prophylaxis to prevent further streptococcal infections, evidence about the best treatment options for SC is lacking [20]. The most common options for symptomatic treatment include antiepileptics (valproic acid and carbamazepine), neuroleptics (pimozide and haloperidol) and monoamine depletors (tetrabenazine). Immunomodulatory treatments with steroids (oral prednisone or intravenous methylprednisolone), intravenous immunoglobulins (IVIg) or plasmapheresis have been utilized, although their role and indications remain controversial [20].

Usually, SC resolves in 1–6 months, although persistence of symptoms for 2 years or more has been reported in up to 50% of patients [16,21]. Relapses affect 13–42% of patients [22,23,24,25] and may occur because of pregnancy (*chorea gravidarum*) or oral contraceptive treatment [21]. Antibiotic prophylaxis is likely to reduce the risk of SC recurrences, although it does not completely prevent them [20]. A higher recurrence rate has been reported in patients with poor regimen adherence [22,23], despite many SC recurrences lacking evidence of streptococcal exposure [26]. The resolution of SC within 6 months could reduce the risk of later recurrences [23].

### 4.2. Systemic Lupus Erythematosus (SLE) and Antiphospholipid Antibody Syndrome (APS)

Chorea is a possible non-thrombotic neurological complication primary APS and SLE (often associated with secondary APS) [27]. APS/SLE-related chorea is more common in children than adults [28] and may be the presenting symptom of the condition [29]. Chorea can be bilateral or unilateral, and it usually develops subacutely [27]. Onset is reported from 5 years of age [30]. Rarely, APS in children and adolescents has been reported in association with acute-onset tics, hemidystonia and parkinsonism [31,32,33]. Psychiatric and behavioral disorders commonly coexist [15]. APS and SLE are the main differential diagnosis of SC in the acute-onset chorea, and antiphospholipid antibodies (aPL) testing is always recommended. Brain MRI can be normal or may show nonspecific findings or basal ganglia and white-matter microvascular lesions [27,34]. CSF is usually normal, but inflammatory changes (mild pleiocytosis, oligoclonal bands, elevated neopterin) can be found [33]. However, no diagnostic criteria for APS/SLE-related chorea exist, and the common occurrence of the MD before other symptoms may prevent the fulfillment of APS or SLE diagnostic criteria, especially in children [27]. Diagnosis relies on aPL positivity and the exclusion of other possible causes. Treatment requires primary or secondary thrombosis prophylaxis and symptomatic therapy for motor symptoms (when needed). Immunosuppressive and immunomodulatory agents are commonly used [29,35,36,37], despite the lack of evidence-based indications [28]. In APS and SLE, MDs may also occur as a consequence of cerebral ischemic events (see below).

### 4.3. Anti-N-methyl-d-aspartate Receptor (NMDAR) and Other Autoimmune Encephalitis (AE)

MDs are a core feature of several AEs [38]. Anti-NMDAR encephalitis is the most common AE, most commonly affecting children and young adults with a female predominance. An association with ovarian teratoma is frequent in young females, while it is rare in children and in males [39]. Onset has been reported as early as in the first months of life [40]. Anti-NMDAR AE may follow a previous herpes simplex virus (HSV) encephalitis [41,42]. Onset in young children usually encompasses speech and sleep disturbances, followed by the appearance of seizures, abnormal movements and behavioral changes. In adolescents, psychiatric symptoms usually predominate at onset [39]. In toddlers, the occurrence of gait disturbances featuring ataxia or freezing of gait has been reported as an early symptom [43,44]. Regardless of the symptoms at onset, the disease usually progresses to include variable combinations of MDs, seizures, behavioral changes, sleep disturbances, reduced consciousness and dysautonomia [39]. The phenomenology of the MD is protean, complex and hardly classifiable. Most patients develop a mixed, hyperkinetic MD frequently featuring dystonia, chorea and stereotypies, and more rarely encompassing tics, myoclonus, ballism, tremor or other stereotyped movements (sometimes described as “tonic” or “clonic perseveration”). The occurrence of orofacial dyskinesia is common and distinctive [45,46,47]. Hypokinetic, namely bradykinetic MDs are rarer and more commonly reported in adolescents, as well as catatonic presentations [48]. Oculomotor disturbances, such as oculogyric crisis and ocular bobbing, are possible [47,49]. MRI is usually normal, but areas of increased fluid-attenuated inversion recovery (FLAIR) signal involving cortical, subcortical or cerebellar regions can be found in about 30% of the cases [39].

Autoimmune Basal Ganglia Encephalitis is the diagnostic label for another clinical syndrome characterized by acquired extrapyramidal movement disorders and neuropsychiatric symptoms, following an infectious illness. Associated features include sleep disturbance and dysautonomia [50]. Negative CSF testing for infectious agents, the presence of oligoclonal bands in CSF and frequent positivity for anti-dopamine D2 receptor autoantibodies corroborate the putative autoimmune pathogenesis of this syndrome [50]. The MD presentation may be both hypokinetic or hyperkinetic. Parkinsonian features may include the variable association of akinesia/bradykinesia, rest tremor and rigidity, while hyperkinetic presentations may feature chorea, ballism, and dystonia [51]. Oculogyric crisis and ocular flutter have been reported in several cases [51]. Brain MRI shows symmetric basal ganglia hyperintensities on T2-weighted images in about half of the cases [51].

Although rarely reported in children [52,53], stiff person spectrum disorders (SPSD) are a possible cause of acquired motor dysfunction whose core symptoms include fluctuating muscle stiffness, superimposed spasms and acquired hyperekplexia [38]. Beside the classic presentation, variant forms may be limited to one limb or associated with other neurological symptoms (SPS-plus). The progressive encephalomyelitis with rigidity and myoclonus (PERM) represents the most severe end of this spectrum [38]. It is a life-threatening disease characterized by brainstem and spinal cord involvement that manifest with hyperekplexia, myoclonic jerks, painful muscle spasms, abnormal eye movements or hemifacial spasm [50]. Associated autoantibodies are directed against glutamic acid decarboxylase (GAD), glycine receptor (GlyR), and less frequently, amphiphysin [38].

Several other, rarer AEs in children may cause MDs. Chorea, dyskinesias and dystonia may be found in anti-LGI1, anti-GABA_A_ and GABA_B_ receptor AEs, usually in the context diffuse or limbic encephalitis [38,54,55]. Myoclonus is a common feature of opsoclonus–myoclonus syndrome (OMS, see below), and may part of the manifestations of AE associated with anti-GABA_B_ receptor or dipeptidyl-peptidase-like protein (DPPX) antibodies [38,54]. Specifically, anti-DPPX-associated AE causes a multifocal disorder with cortical, brainstem, cerebellar, spinal cord and autonomic nervous system involvement. Frequent features include encephalopathy, abnormal eye movements, dysautonomia, gastrointestinal dismotility, cerebellar ataxia and myoclonus, with clinical overlap with PERM and OMS [56]. MDs associated with anti-DPPX AE also include parkinsonism, chorea, dystonia and tremor [56]. Diarrhea with profound weight loss and other gastrointestinal symptoms are a characteristic symptom of the initial stages of this disease [38].

AEs are susceptible to immune-suppressive treatments, including steroids, IVIg, rituximab, cyclophosphamide and plasmapheresis [57].

### 4.4. Acute Disseminated Encephalopathy (ADEM)

Although rarely, MDs may be the presenting or prominent symptom of pediatric demyelinating diseases, especially ADEM [58]. Patients with ADEM may feature choreoathetosis, hemidystonia, hemichorea, facial dyskinesias, segmental myoclonus and paroxysmal hemidystonia [59,60,61]. In addition, complex MD featuring dystonia and abnormal eye movements has been reported in a toddler with anti-myelin oligodendrocyte glycoprotein (MOG)-associated ADEM [62].

### 4.5. Opsoclonus–Myoclonus Syndrome (OMS) and Paraneoplastic MDs

OMS is a rare autoimmune MD that typically affects young children (mean age 18–24 months [63,64]), but that may occur throughout life from infancy to elderly [65]. The disease usually begins with behavioral abnormalities (excessive irritability and sleep disturbances) and developmental stagnation [65]. Later, ataxia and/or trunk and limb myoclonus appear acutely or subacutely (over hours to weeks). The association of opsoclonus—the most distinctive manifestation—is not universal [65]. Pediatric OMS is a paraneoplastic phenomenon related to neuroblastoma in about 50% of the cases [66], and rarer associations with ganglioneuroma, hepatoblastoma are possible [65]. In adolescents (especially females), the association of OMS, additional signs (ophthalmoparesis, dysarthria, altered consciousness) and ovarian teratoma (without anti-NMDAr antibodies) has been described as a distinctive brainstem–cerebellar syndrome [38,67]. Therefore, in-depth work-up for occult tumor detection is mandatory, including the testing of homovanillic acid and vanillylmandelic acid urine levels and chest, abdominal, and pelvic CT or MRI. I^123^-metaiodobenzylguanidine scan or ^18^F- fluorodeoxyglucose PET are second-level investigations, useful if CT or MRI are unrevealing or to detect metastatic involvement [65]. If negative, tumor screening must be repeated during follow-up, since OMS may precede the diagnosis of neuroblastoma [68]. Brain MRI is typically normal. CSF testing may reveal the presence of inflammatory markers (lymphocytic pleocytosis, elevated neopterins and oligoclonal bands). Nonparaneoplastic OMS in children is usually considered to have a para- or post-infectious origin, or it is labelled as idiopathic. Despite the plethora of triggering infectious agents reported [69,70,71,72,73,74,75,76,77,78,79], no consistent pathogen has emerged. In addition, the triggering infection may be difficult to demonstrate, and clinical features and immunological markers are not helpful in differentiating paraneoplastic from non-paraneoplastic cases [80]. Specific autoantibodies against intracellular (anti-Hu, anti-CV2/CRMP5, anti-Ma2 [81,82,83,84]) or surface antigens (such as glutamate receptor δ2 [85,86,87,88,89]) have been detected, but their diagnostic value in children is still uncertain [65,90]. The association of OMS with established pathogenic autoantibodies (anti-NMDAR, GABA_A_ and GABA_B_ receptor, DPPX, GAD, GlyR [54,81,91,92,93]) is anecdotal or limited to adult forms. Besides tumor management, the treatment of OMS relies on immunomodulatory therapies, including adrenocorticotropic hormone (ACTH), steroids, and IVIg [65].

Exceptionally, acute-onset chorea has been described in association with cardiac fibroelastoma, in a putative paraneoplastic syndrome [94,95].

## 5. Infectious and Para-Infectious Disorders

Beside their trigger role in multiple immune-mediated disorders, infectious agents may cause MDs by direct invasion of the central nervous system (CNS) [96]. Nevertheless, it is sometimes difficult to discriminate the infectious from the immune-mediated mechanism, and both processes may occur simultaneously or in succession. In this section, we discuss MDs occurring in association with demonstrable CNS infections or in the context of systemic infectious illnesses.

### 5.1. Viral Infections

In the context of viral encephalitis, some agents have been reported to frequently cause MDs [96], because of a specific tropism for the basal ganglia, thalami and brainstem [97]. In children, Epstein–Bar virus (EBV) encephalitis may cause parkinsonism [98,99], choreoathetosis may be seen in the acute phase of influenza A, EBV and HHV-6 encephalitis [100,101,102], and tics have been described in post-varicella encephalitis [103]. In endemic countries, Japanese and Dengue encephalitis are typically associated with parkinsonism and/or dystonia, with the characteristic occurrence of oromandibular dystonia [97,104,105,106]. By converse, MDs are rarely associated with HSV encephalitis, and the numerous reports of post-HSV encephalitis relapses associated with chorea and other MDs are actually due to subsequent anti-NMDAr AE [41,42,107]. Although rare, HIV-encephalopathy is a possible cause of acute-onset chorea, oro-facial dyskinesias, bradykinesia and dystonia (sometimes mimicking anti-NMDAr AE [108,109,110]).

### 5.2. Bacterial Infections

Children with tuberculous meningitis frequently develop MDs as a consequence of inflammation, essudate, hydrocephalus, and vascular lesions in the basal ganglia, diencephalon and brainstem [111]. Chorea is probably the most common MD in young children [111], but tremor and dystonia may occur [111,112], sometimes progressing to *status dystonicus* [113]. Chorea, athetosis and ballismus may also occur in the acute stage of bacterial meningitis due to *H. influenza*, *S. pneumoniae* or *N. meningitidis* [114]. In one case, tourettism has been reported as a possible presentation of neuroborreliosis [115].

### 5.3. Protozoal, Fungal and Helmintic Infections

Cerebral toxoplasmosis abscesses, mostly affecting immunocompromised patients, usually localize to the basal ganglia, thalami and midbrain [96] and may cause chorea and dystonia [16,116], although it is extremely rare in children. Similarly, cryptococcal abscess are a potential alternative cause [16]. In endemic areas, neurocysticercosis has been reported to cause ballismus, chorea and dystonia [117,118,119].

### 5.4. Infectious and Post-Infectious Bilateral Striatal Necrosis (BSN)

BSN is a pathologically and radiologically defined condition characterized by the subsequent appearance of neostriatal swelling (that appears on brain MRI as hyperintensity of putamina and caudate nuclei on T2-weighted images) followed by progressive degeneration with cellular necrosis and cavitation (corresponding to T1-hypointensity and hypo-hyperintensity on fluid inversion recovery images) [120]. These findings may be encountered in both acquired and genetic conditions, with many cases having been linked to infectious disease [120,121]. Usually, the disease presents acutely with the signs of the systemic illness, while manifestations of CNS involvement may develop along with the intercurrent illness or shortly after its resolution [120,122,123,124,125,126]. Neurological symptoms usually include dystonia and/or parkinsonism, variably associated with encephalopathy, seizures, ataxia or pyramidal signs [120]. The most commonly associated infectious agent is *Mycoplasma pneumonia* [122,123,126], but several other possible causes have been reported—such as GABHS, HHV-6 or measles [124,125,127,128,129]. Laboratory investigations do not always clarify if pathogenesis is supported by the infection or immune-mediated mechanisms [120]. The disease is typically monophasic and non-progressive, followed by gradual—although partial—recovery [120]. The occurrence of subsequent relapses, progressive deterioration, or pre-existing neurological symptoms should lead one to consider a metabolic etiology (see below).

### 5.5. Acute Necrotizing Encephalopathy (ANEC)

ANEC is a rare para-infectious disease, triggered by different viral infections [130]. Initial reports from Japanese and Taiwanese children [131] suggested a geographical predilection for eastern Asia, but recent reports have shown that ANEC has a global distribution [130]. The disease develops in previously healthy children after common viral illnesses, especially influenza virus and HHV-6 [130]. The signs of the viral illness dominate the prodromal stage. Later, the disease progresses with the occurrence of encephalopathy, hyperpyrexia, signs of liver dysfunction and uremia [131], that may progress into multiorgan failure and disseminated intravascular coagulation (DIC) [130]. Neurological signs include coma, seizures, and focal deficits. Children with ANEC may feature dystonia, parkinsonism and chorea [2,3]. The disease course is fulminant, and the outcome may vary from complete recovery after a recovery stage to severe forms with high mortality or significant neurological disability [130]. The pathological and radiological hallmark of the disease is the occurrence of multifocal, bilateral and symmetrical brain lesions that invariably involve the thalami and usually extend to brainstem, cerebral white matter, and cerebellum [130], with occasional involvement of the spinal cord [132]. ANEC pathogenesis is unclear. Although viral agents may sometimes be detected on CSF, the currently predominant hypothesis is that ANEC results from an exaggerated proinflammatory response leading to a cytokine storm. On this basis, immunomodulatory therapy with glucocorticoids, IVIg and plasmapheresis can be tried, but evidence of efficacy is lacking [130,133]. Although ANEC is usually an isolated and sporadic disease, recurrent and familiar cases are possible and can be due to *RANBP2* gene mutations [130].

## 6. Vascular Diseases

Unlike in adults, cerebrovascular disease is a rare cause of acute MDs in children [3,4,5]. Nevertheless, basal ganglia strokes may cause unilateral chorea or ballismus [3,4,134].

Chorea, ballismus and dyskinesia can be seen in Moyamoya disease [135,136], a chronic cerebral vasculopathy that provokes uni- or bilateral progressive stenosis of the terminal portion of the internal carotid artery, associated with the development of a fine collateral network at the base of the brain [137]. Abnormal movements may have paroxysmal or highly fluctuating course [135,136], precipitated by hyperventilation or emotional stress, probably as a consequence of hypoperfusion of the basal ganglia [16].

“Post-pump chorea” is an infrequent complication of cardiopulmonary bypass that occurs within 2 weeks from surgery [138]. Chorea mostly involves the limbs, mouth, tongue and face and is usually associated with variable degrees of encephalopathy [16]. Its occurrence may be favorited by deep hypothermia, prolonged extracorporeal circulation time, and variability in blood pH and PaCO_2_ during surgery [139]. Neuroimaging studies are usually normal or show unspecific findings, and the pathophysiology remains unclear [139]. Chorea can be transient or persistent, with an increased risk for severe and persistent forms in older children compared to infants [139].

## 7. Drug-Induced and Toxic Movement Disorders

Acute MDs are a potential side effect of numerous drugs. Besides prescribed treatment, children are exposed to the risk of inadvertent or unwise use of drugs and several toxic agents [140].

### 7.1. Acute Dystonic Rreactions

Acute dystonic reactions (ADR) usually occur after exposure to dopamine receptor blocking agents (DRBAs), such as neuroleptics and antiemetics (illustrative case 2, Supplementary Material) [140]. Typically, ADRs involve the head, face and neck, causing retrocollis, opistothonus, trismus, tongue protrusion or oculogyric crisis. Extremities are less frequently involved [141]. Rarely, laryngeal spasm represents a life-threatening form [141,142]. ADR usually develops within 5 days from assumption of the offending drug, and children are at increased risk compared to adults [140]. The risk is higher with typical, potent DRBA (such as haloperidol), but drugs inducing a less potent dopaminergic blockade may also cause ADR, including atypical antipsychotics (such as aripiprazole), antiemetics (such as metochlopramide) or selective serotonin reuptake inhibitors (SSRIs, such as citalopram or escitalopram), especially in children [141,143,144,145]. In addition, ADRs have been reported after exposure to methylphenidate or other psychostimulants in children treated with (or shortly after the withdrawal of) DRBA [146,147,148], suggesting that dopaminergic drugs may increase the risk of ADRs in patients exposed to DRBA. Nevertheless, methylphenidate may cause several dyskinetic reactions, including focal dystonia, also in DRBA-naïve patients [149,150,151,152,153]. Treatment with anticholinergic drugs may be beneficial [141].

### 7.2. Neuroleptic Malignant Syndrome

Neuroleptic malignant syndrome (NMS) is a drug-induced condition that usually develops within one month from the beginning or an increase in dosage of a DRBA, or (exceptionally in children) after abrupt the discontinuation of dopaminergic treatments [140]. The risk of NMS is greater for typical than for atypical neuroleptics. Rarely, NMS may be induced by antiemetic DRBAs, SSRIs, lithium, tryclic antidepressants (TCAs) or metylphenidate [140,154,155,156,157,158,159]. NMS manifests with the acute appearance of hypertermia, altered consciousness, severe rigidity, autonomic instability and hyperCKemia [140,160]. Associated MDs may include dystonia, chorea, parkinsonism, oro-facial dyskinesia and oculogyric crisis [140]. NMS is a life-threatening emergency, potentially evolving into cardiac and renal failure, respiratory disturbances and DIC [140]. Treatment requires the discontinuation of the offending drug, supportive measures, and specific therapies such as bromocriptine (a dopamine agonist), dantrolene and amantadine. Nevertheless, evidence of efficacy for these latter treatments is lacking [140].

### 7.3. Serotonin Syndrome

Serotonin syndrome (SS) develops after exposure to one or (more commonly) multiple serotonergic drugs [140]. SS may be induced by the overdose or interaction of a variety of compounds with intrinsic serotonergic activity or inhibiting serotonergic drug metabolism, including antidepressants (such as SSRIs, TCAs, monoaminoxidase inhibitors), psychostimulants (amphetamines, methylphenidate), drugs of abuse (cocaine, MDMA), antibiotics or antivirals (linezolid, erythromycin, ritonavir), antimigraine drugs (triptans, ergotamines), and nutraceuticals [140]. SS usually occurs within 24 h from exposure to the offending agents, presenting with agitation, mental status changes, myoclonus, hyperreflexia, clonus, tremor, ocular flutter, dry mouth, fever and dysautonomia [161]. Treatment relies on the discontinuation of serotonergic drugs and supportive measures. The serotonin antagonist cyproheptadine is often used, but evidence of efficacy is poor [161,162].

### 7.4. Other Iatrogenic Movement Disorders

Beside the above-cited methylphenidate-induced dyskinesias, several other drugs have the potential to cause acute-onset abnormal movements. Chorea and dyskinesia may be acutely induced in children by other psychostimulants [163], ACTH, vigabatrin, theophylline, aminophylline, midazolam, phenytoin and other anticonvulsants [164,165,166,167,168,169,170,171,172], as well as by the rapid discontinuation of intravenous midazolam [173,174]. Although better reported in adults, anticholinergics, L-DOPA, dopamine-agonists, opioids, TCAs, baclofen and lithium may also cause chorea [16,175,176,177,178,179,180,181,182]. Oral contraceptives may cause chorea in adolescent girls, especially in patients with previous SC [183,184]. Myoclonus may be induced by opioids, SSRIs, etomidate, carbamazepine and its derivatives, ketamine and analgesic withdrawal [3,178,185,186,187,188]. Carbamazepine may also cause tics [189]. Acute-onset parkinsonism or chorea may be observed in patients exposed to amphotericin B and cytosine arabinoside [3,190,191,192,193].

### 7.5. Systemic Intoxications

Although rarely reported in children, carbon monoxide poisoning may cause parkinsonism, and less frequently chorea, athetosis, dystonia and tremor, sometimes with delayed onset from intoxication [194]. Mercury intoxication may cause tremor [4], lead poisoning may induce myoclonus [195], and overdoses of decoctions containing different medicinal herbs may cause encephalopathy with dystonia [196].

## 8. Inborn Errors of Metabolism and Genetic Disorders

Acute-onset MDs are a possible presentation of various genetic disorders in children, mostly in the setting of the acute decompensation of IEM, often with accompanying encephalopathy [197].

### 8.1. Organic Acidurias

Glutaric aciduria type I typically presents within 3 years of age with acute encephalopathic crises associated with generalized dystonia and dyskinesia. Common triggers include intercurrent illnesses or minor head trauma. MRI findings include BSN, frontotemporal atrophy, white matter alterations, pseudocysts, and chronic subdural hematomas [120,198]. Treatment relies on a low-lysine and carbohydrate-enriched diet with carnitine supplementation [198]. Other organic acidurias that may present with acute encephalopathy with dystonia, dyskinesias and choreoathetosis include maple syrup urine disease, propionic, 3-methylglutaconic and methylmalonic acidemias, and cobalamin C defects [197,198,199]. Early diagnosis and the initiation of appropriate dietary and pharmacologic interventions prevents further neurological deterioration.

### 8.2. Mitochondrial Disorders

Leigh syndrome (LS) is a subacute necrotizing encephalopathy typically manifesting in infancy or early childhood, triggered by infections, starvation and other catabolic states [120,200]. Clinical presentation usually includes developmental regression with encephalopathy, dystonia, parkinsonism, ataxia and pyramidal signs, possibly associated with signs of muscular, neuropathic, renal, or cardiac involvement [120]. More rarely, LS may cause choreoathetosis. Radiologically, LS is characterized by symmetrical and bilateral lesions variably involving the basal ganglia, thalami, cerebral cortex, white matter, cerebellum, brainstem, and spinal cord [120,201], and isolated BSN is a possible presentation [120]. LS is genetically heterogeneous, being caused by a wide range of mutations in mitochondrial and nuclear genes impairing energy production [201,202]. Nevertheless, the list of genes causing LS or LS-like presentations by different molecular mechanisms is permanently expanding [202]. Among them, Biotin–thiamine-responsive basal ganglia disease (BTBGD) is a potentially treatable disease due to defects in the thiamine transporter 2, encoded by the *SLC19A3* gene. BTBGD usually presents recurrent episodes of acute or subacute encephalopathy in infancy, often triggered by catabolic states, featuring seizures, confusion, ophthalmoplegia and dystonia. Supplementation with biotin and thiamin may considerably improve the outcome [198]. Other IEMs that may cause acute encephalopathy with MDs present with combined features of organic acidurias and mitochondrial disorders, including succinate-CoA ligase deficiency, ethylmalonic encephalopathy, and valine metabolism defects [120,198].

### 8.3. Other Genetic Disorders

Rapid-onset dystonia-parkinsonism due to *ATP1A3* gene mutations presents with acute or subacute dystonia associated with minor parkinsonian features. Typically, anatomical distribution is asymmetrical, with prominent bulbar signs and a rostro-caudal gradient of involvement [203]. Symptoms usually progress over hours to weeks and remain stable thereafter. Onset may range from infancy to adulthood, and provoking factors such as emotions, infections, trauma, or pregnancy are usually recognizable. In addition, acute-onset choreoathetosis, dystonia and dyskinesia may be encountered in other *ATP1A3*-related phenotypes, such as recurrent encephalopathy with cerebellar ataxia and CAPOS syndrome (cerebellar ataxia, areflexia, pes cavus, optic atrophy, and sensorineural hearing loss) [203,204].

Rarely—in its juvenile dystonic form—Wilson’s disease may have subacute onset with rapid deterioration [205,206]. In addition, acute neurologic deterioration may occur after initiation on penicillamine chelating therapy [198].

Besides infectious and metabolic causes, an important differential diagnosis of acute-onset MDs with bilateral striatal lesions is *ADAR1*-related disease, a genetic interferonopathy that may present with acute dystonia or choreoathetosis and developmental regression following an infectious trigger (illustrative case 3, Supplementary Material) [207].

Although rarely, neurodegenerative disorders such as ceroido lipofuscinosis, may present with acutely or subacutely evolving MDs, usually after an insidious onset of neurological signs [4,5,208].

## 9. Metabolic and Endocrine Disorders

Although uncommon in children, acute MDs may result from metabolic and endocrine imbalance induced by a variety of disorders. Acute hemichorea–hemiballismus induced by non-ketotic hyperglycemia is a possible complication of type 1 diabetes—although more common in type 2 [96,209,210]. Tremors, myoclonus and asterixis may be seen in acute hepatic and uremic encephalopathy [211]. Electrolyte disturbances may cause a wide range of MDs: hypernatriemia may cause tremor, chorea, or myoclonus, hypercalcemia may cause myoclonus and hyperreflexia, and hypomagnesemia induces tremor, chorea, myoclonus and exaggerated startles [212]. Thyrotoxicosis can cause tremor and has been rarely reported to induce chorea [213]. In addition, nutritional deficiencies such as vitamin B12 may cause tremors [4].

## 10. Paroxysmal Sympathetic Hyperactivity

Paroxysmal sympathetic hyperactivity (PSH)—also known as paroxysmal autonomic instability with dystonia—is usually a complication of severe acquired brain injury (ABI), regardless of etiology [214,215]. It manifests with intermittent agitation, dysautonomia (diaphoresis, hyperthermia, hypertension, tachycardia, and tachypnea), rigidity and dystonic posturing [2,214]. Dystonia usually features axial extensor postures with opistothonus or decerebrate/decorticate posturing, but hemydystonia is possible [214,216]. It has been originally described (under different terms) as occurring within one week from severe traumatic or anoxic ABI, but it can be due to tumors, intracranial hemorrhage, infections or hydrocephalus [214,215,217,218,219]. It is more likely to occur in the case of diffuse axonal injury or brainstem–diencephalic lesions [214]. More rarely, presentations similar to PSH have been reported in genetic syndromes, such as Rett’s or Down’s [2,220,221].

The clinical course may fluctuate, with persistence over weeks or months [214]. PSH shows overlapping features with NMS and *status dystonicus*, but rhabdomyolysis is infrequent and dysautonomia is prominent [8,214]. In addition, PSH shares features and may coexist with other complications of severely ill patients, including sepsis, drug withdrawal, delirium, or pain [214]. Treatment is largely supportive. Since an excessive sympathetic activity is implied, propranolol, clonidine and other adrenergic inhibitors may be helpful [8,214,222]. To ensure sedation and treat motor symptoms, opioids, benzodiazepines and gabapentin may be used [8,214]. In analogy with NMS, bromocriptine may be beneficial in some cases [214].

## 11. Tics Disorders and Tourette Syndrome

Acute vocal and motor tics have been reported as the most common acute MD in children presenting to pediatric emergency departments [5]. Although tic disorders usually have an insidious onset between in school-aged children [223], acute appearance or sudden exacerbations leading to medical attention are not uncommon [5]. The course of tics is spontaneously fluctuating [224], but acute and sustained “bouts” of tics may be provoked by stressful life events, fatigue, intercurrent medical issues, or drugs (especially psychostimulants and serotonergic agents). [225,226,227,228]. In rare cases, uncontrolled and violent movements (especially of the head and neck) may result in tic-related injuries [229], or exacerbations may be so severe as to shape a non-suppressible, restless tic succession lasting for hours, sometimes referred as “tics status” [226].

When tics acutely occur in children that are not known to suffer from tic disorders, investigations are usually unnecessary [223]. Observation and careful history are sufficient to identify tics: their stereotyped nature (configuring a “tic repertoire”), the occurrence of premonitory sensory experiences (relieved by the tics) and the possibility to suppress tics for brief time intervals are specific features of this MD [223]. Even in acute presentations, a previous insidious history of mild tics often emerges.

In adolescents with an atypical onset of tics after puberty, the most difficult differential diagnoses are functional tics (see below). From the late 1990s, it has been strongly debated whether a subset of children presenting with acute-onset tics and/or obsessive-compulsive disorder after exposure to GABHS infections may have an underlying autoimmune disease (in analogy with SC) [230]. These children have been proposed to configure a separate entity—referred as pediatric autoimmune neuropsychiatric disorders associated with streptococcal infections (PANDAS), characterized by a relapsing–remitting course attributed to re-infections and by a positive response to antibiotic or immunomodulatory treatments [231]. Despite extensive research and controversy, biological studies failed to convincingly demonstrate an immune basis, evidence of efficacy of antibiotics and immunotherapy is poor and recent studies show that GABHS infections are not trigger factors for tic exacerbations [230,232]. As a result, the search for evidence of streptococcal exposure in children with tics should be discouraged.

## 12. Functional Movement Disorders

Functional MDs have been reported to account for 4.3–23% of all acute MDs in children and adolescents, representing one of the most common causes [233]. Tremor is the most common functional MD (illustrative case 4, Supplementary Material), followed by myoclonus, dystonia and tics, and the combination of multiple MDs is frequent [3]. Recently, the abrupt, explosive onset of tics and tic-like movements in adolescents (with a female predominance) has been described as an unusual presentation of functional tics, whose frequency could have been raised by the COVID-19 pandemic and related lockdown measures [234].

Diagnosis relies on the demonstration of specific features on history and examination. First, functional MDs often present with abrupt and dramatic onset [233]. Second, symptoms and their severity tend to vary, and disability is often selective or inconsistent [10,233]. Distractibility (namely, the disappearance or reduction in the severity of the symptoms when the patient is unobserved or distracted by a cognitive task) is a strong argument in favor of a functional etiology. Similarly, the entrainment phenomenon can be actively searched in tremor and myoclonus: it consists in the synchronization of the involuntary movement to an externally imposed rhythm, and strongly suggests a functional MD [10]. In addition, several features can make functional phenomenology incongruent with an organic origin. For instance, dystonia lacks sensory tricks or overflow, dystonic posturing is usually fixed instead of mobile, and pain can be a prominent feature. Tics lack a premonitory urge and cannot be voluntarily suppressed, while tremor may emerge in a distant, previously unaffected body segment after constraining the originally tremulous body part (whack-a-mole sign, see [233] for a detailed list of incongruent features). Chronic underlying medical, psychiatric or neurologic conditions may be present, and psychosocial or physical stressors are frequent triggers [3].

Despite these positive features, sometimes, functional MDs can be difficult to diagnose and both functional and organic MDs may coexist in the same patient [10].

## 13. Conclusions

**Figure 2:**
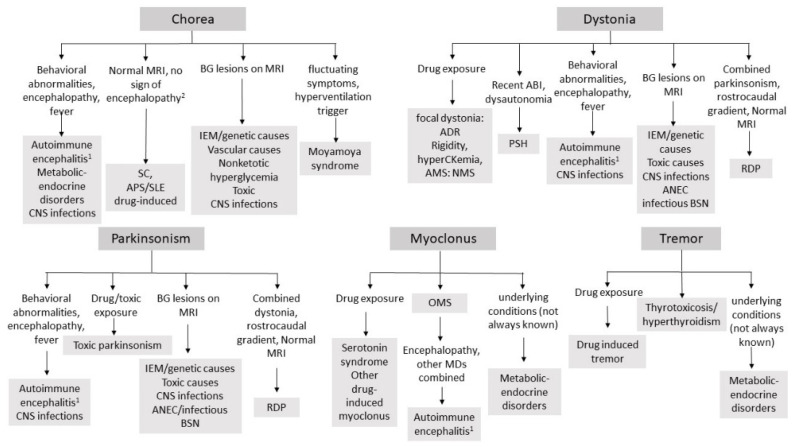
Essential diagnostic algorithm for acute-onset MDs, according with the predominant phenomenology. The list of suggested etiologies is not exhaustive. ADR: acute dystonic reaction; AMS: altered mental status; ANEC: acute necrotizing encephalopathy; APS: antiphospholipid syndrome; BSN: bilateral striatal necrosis; CNS: central nervous system; IEM: inborn errors of metabolism; MRI: magnetic resonance imaging, NMS; neuroleptic malignant syndrome, OMS: opsoclonus–myoclonus syndrome; PSH: paroxysmal sympathetic hyperactivity; RDP: raid-onset dystonia-parkinsonism.

In this review, we summarized common and rare causes of acute-onset MDs in children and adolescents (see Supplementary Table S1 for a summary). In their acute presentation, MDs may represent the prominent symptom or an important diagnostic clue in a broader constellation of neurological and extraneurological signs. For this reason, the diagnostic approach relies on the definition of the overall clinical syndrome (Figure 1) and on the recognition of the prominent MD phenomenology (Figure 2). Appropriate management relies on the identification of the underlying disorder. Acute MDs are mostly due to autoimmune and inflammatory diseases, and their prompt recognition may have a significant impact on outcome [3]. Similarly, the timeliness of treatment initiation in treatable IEM is pivotal to avoid ongoing neurological deterioration [198]. Consequently, MD emergencies emerge as a pivotal part of every child neurologist’s wealth of knowledge.

## Figures and Tables

**Table 1:** Suggested investigations for acute-onset MD, according with the predominant phenomenology (a non-exhaustive list).

**Chorea**
*First-line tests* throat swab culture, ASO and antiDNAse titer, cardiac US, EKG, CRP, ESR Routine blood test Neuroimaging
*Second-line tests:* aPL (anti GP2 IgG and IgM, anti cardiolipin IgG and IgM, LAC assay), to be always performed if chorea does not fulfil Jones’ criteria for rheumatic fever, ANA, ENA, complement fractions (C3–C4), consider joint US if signs of arthritis TSH/fT4 If signs of encephalitis ^i^/encephalopathy: EEG, CSF sampling for cell count, proteins, CSF culture, PCR for neurotropic viruses, consider OCB, specific aAb testing on CSF and serum (NMDAR, D2R) Consider studies for IEM ^1^ if suggestive findings on MRI, young age, encephalopathy induced by catabolic states or high protein intake Consider MRA to exclude vasculopathy (especially if fluctuating symptoms or in presence of vascular lesions)
**Dystonia**
*First-line tests* Exclude drug/toxic exposure (toxic screening if needed) Routine blood test Neuroimaging
*Second-line tests:* If signs of encephalitis/encephalopathy: EEG, CSF sampling for cell count, proteins, CSF culture, PCR for neurotropic viruses, consider OCB, specific aAb testing on CSF and serum (NMDAR, D2R) Consider screening for IEM ^1^ (including ceruloplasmin/copper studies), especially if suggestive MRI findings. If severe dystonia: CPK, myoglobin, creatinine, urea.
**Myoclonus**
*First-line tests* Tumor screening (HVA/VMA urine levels, pelvis/abdomen/chest CT or MRI, MIBG scan). Exclude metabolic or endocrine disorders (uremia/hepatic encephalopathy). Exclude drug/toxic exposure (toxic screening if needed) Routine blood test Neuroimaging
*Second-line tests:* If rigidity, ataxia, or signs of encephalopathy: EEG, CSF sampling for OCB, cell count, proteins and specific aAb testing on CSF and serum (anti-DPPX, GlyR, GAD, amphysin). Consider work-up for IEM ^1^, according with MRI findings, especially if young age
**Parkinsonism**
*First-line tests* Routine blood test Neuroimaging Exclude drug/toxic exposure (toxic screening if needed)
*Second-line tests:* If signs of encephalitis/encephalopathy/behavioral abnormalities ^i^: EEG, CSF sampling for OCB, cell count, proteins, CSF culture, PCR for neurotropic viruses, specific aAb testing on CSF and serum (NMDAR, D2R). consider work-up for IEM ^1^ (including ceruloplasmin/copper studies), especially if suggestive MRI findings
**Tremor**
*First-line tests* Exclude toxic causes (drug/toxins) and metabolic/endocrine disorders: electrolyte imbalances, TSH/fT4 testing; blood glucose levels; toxic screening (if needed) Consider Neuroimaging
*Second-line tests:* If signs of encephalopathy/encephalopathy: EEG, CSF sampling for OCB, cell count, proteins, CSF culture, PCR for neurotropic viruses, specific aAb testing on CSF and serum (NMDAR, D2R). Consider Screening for IEM ^1^ (including ceruloplasmin/copper studies), according with MRI finding

^i^ If signs of encephalitis, exclusion of infections of the central nervous system should be prioritized. ^1^ Suggested IEM screening: ABG, ammonium and lactate levels (blood/CSF), urinary organic acids, plasma acylcarnitines, plasma aminoacids, homocysteine levels, ketones. If suspicion of mitochondrial disorders: magnetic resonance spectroscopy, pyruvate, thiamine, biotin; consider muscle biopsy for histology and respiratory chain complexes activity, Western blot for detection of mtDNA depletion. Abbreviations: ABG: arterial blood gas, aPL: antiphospholipid autoantibodies, ASO: anti-streptolysin O; US: ultrasonography, EEG: electroencephalogram, EKG: electrocardiogram, CRP: C-reactive protein, ESR: erythrocyte sedimentation rate; GP2: glycoprotein 2, LAC: Lupus anticoagulant; CSF: cerebrospinal fluid; OCB: oligoclonal bands; IEM: inborn errors of metabolism, MRA: magnetic resonance angiography; CPK: creatine phosphokinase; HVA: homovanillic acid; VMA: vanillymandelic acid.

## Data Availability

Not applicable.

## Supplementary Materials

The following are available online at https://www.mdpi.com/article/10.3390/jcm10122671/s1, Table S1: MD phenomenology and relevant neuroimaging and biochemical findings in acute-onset MD in children and adolescents.
