# Supplementary material for: Acute Movement Disorders in Childhood
Source: J Clin Med. 2021 Jun 17;10(12):2671. doi: 10.3390/jcm10122671 (PMC8234395; doi:10.3390/jcm10122671)
Supplement: Supplementary file 1 [file jcm-10-02671-s001.zip › jcm-1251317-supplementary.pdf]

Acute movement disorders in childhood

Giacomo Garone[1,2], Federica Graziola[1,3], Melissa Grasso[1], Alessandro Capuano[1*]

[1]Neurology Unit, Department of Neurosciences, Bambino Gesù Children's Hospital, Rome, Italy,

[2]University Department of Pediatrics, Bambino Gesù Children's Hospital, University of Rome Tor Vergata, Rome, Italy

[3]University of Rome Tor Vergata, Rome, Italy

Supplementary Table

	Chorea	Myoclonus	Dystonia	Parkinsonism	Tremor	MRI	Recommended Investigations and relevant findings
**Immune-mediated disorders**							
Sydenham Chorea	++					Usually normal, helpful to rule out different causes	positive GABHS throat swab culture, raised ASO and antiDNAse titer, raised CRP/ESR, signs of rheumatic carditis on cardiac US and EKG
SLE/APS	++		(+)	(+)		Usually normal or unspecific findings	positive aPL (anti GP2 IgG and IgM, anti cardiolipin IgG and IgM, LAC assay), +/- positive ANA and ENA, hypocomplementemia
Anti-NMDAr AE	+	+	+	+	(+)	normal or FLAIR hyperintensities on cerebral cortex, hippocampus, basal ganglia, WM	CSF lymphocytic pleocytosis or oligoclonal bands (not constant); positive anti-NMDAr aAb on serum and CSF, abnormal EEG
Basal Ganglia encephalitis	+		++	++	(+)	BG bilateral T2W hyperintensities (50% of cases)	CSF lymphocytic pleocytosis or oligoclonal bands (not constant); raised CSF neopterins, positive anti-D2R aAb on serum and CS
Demyelinating disorders	(+)	(+)	(+)			Diffuse, asymmetrical lesions of cerebral WM, Deep GM lesions (BG and thalami)	CSF or normal lymphocytic pleocytosis, positive OCB, possible anti-MOG-IgG positivity in MOG-related ADEM)
Opsoclonus-Myoclonus-syndrome		++				Usually normal, helpful to rule out different causes	In paraneplastic OMS: raised HVA/VMA; tumor mass on pelvis/abdomen/chest CT/MRI. CF sampling may show inflammatory alterations. Aab testing is not routinely recommended in children

Paraneoplastic Chorea	++					Usually normal, helpful to rule out different causes	fibroelastoma on cardiac US
**Infectious and post-infectious disorders**							
Viral encephalitis	+		++	++		Variable according with the viral agent (common involvement of BG, thalami, brainstem in MD-causing encephalitis)	CSF lymphocytic pleocytosis, positive PCR testing for viral agents
Bacterial meningitis	+					Variable:	CSF mononuclear pleocytosis, hypoglycorrhachia, positive CSF cultures
Tubercular Meningitis	++		++	(+)	+	BG and brainstem infarcts, hydrocephalus, basal ganglia tuberculomas	CSF: raised proteins, normal glucose, lymphocyte predominant pleocytosis. Signs of TB on chest radiography
Post-infectious Bilateral Striatal Lesions	+		++	++		BSN	CSF: raised neopterins, mild pleocytosis, raised protein levels. Positive microbiological tests (e.g. troath swab for M. pneumoniae), sometimes detectable on CSF
Acute necrotizing encephalopathy	+		+	+		bilateral and symmetrical lesions of thalami + brainstem, WM, cerebellum, sometimes spinal cord	uremia and hepatic dysfunction (possibly evolving into MOF/DIC), raised inflammatory markers. Positive microbiological test according with the triggering illness (throat/nasal swabs, stool culture…). Possible detection of infectious agents on CSF
**Vascular Diseases**							
Stroke	+					BG ischemic or hemorrhagic lesions (in MD-causing strokes).	According with the predisposing cause (if any)
Moyamoya syndrome	+					ICA uni- or bilateral stenosis, collateral network (visible on MRA and angiography). Possible presence of watershed infarcts or strokes. SPECT may demonstrate hypoperfusion (sometimes with acetazolamide test). Consider investigation for predisposing conditions (e.g. SCA)	
Post-pump chorea	++					Normal, possible cerebral atrophy (helpful to exclude vascular lesions)	
Drug-induced movement disorders							
Acute dystonic reactions			++			Normal (helpful if relationship with the offending drug is uncertain or incongruent with the clinical picture)	Consider Toxic screening (urine/blood) if toxic agent is unknown

Neuroleptic malignant syndrome			++			Normal	massive hyperCKemia (possible myoglobinuria with renal failure)
Serotonine Syndrome		++				Normal	HyperCKemia is possible (milder than in NMS)
**Organic acidurias**[1]							
Glutaric aciduria Type 1	+		++	(+)		BSN, frontotemporal atrophy, white matter alterations, pseudocysts, and chronic subdural hematomas	ketonemia, metabolic acidosis, glutaricaciduria
Propionic acidemia	++		++			BG infarcts	metabolic acidosis, hyperammonia, hyperglycine, hypoglycemia, low carnitine, raised propionate, 3-hydroxypropionic acid and 3-methylcitric acid
Methylmalonic acidemia	+		+			GP hyperintensities (T2W), BG strokes, delayed myelination	Metabolic ketoacidosis, hyperammonia, hyperglycinemia, reduced plasma free and total carnitine, methymalonicaciduria
MSUD	+		+			Brain edema, WM abnormalities	Ketoacidosis, hypoglycemia, raised leucine, isoleucine, valine, ketoaciduria (alpha-keto isocaproate, alpha-keto-beta methylisovalerate, alpha-keto isovalerate)
**Mitochondrial Disorders**[1]							
Leigh syndrome (or Leigh-like)	+		++	++		BG, WM, cerebellum and brainstem T2W hyperintensities, BSN, raised lactate on MRS	raised blood and CSF lactate, reduced activity of RCC
BTBGD			++	+		striatal hyperintensities, raised lactate on MRS	lactic acidosis, raised CSF lactate, reduced CSF free thiamine
**Other Genetic conditions**							
Rapid-onset Dystonia Parkinsonism			++	++		usually normal	No specific findings
Wilson disease			+			T2W hyperintensity on putamina and caudate, cerebellum, brainstem, WM (Face of the panda)	Ceruloplasmin, 24-hour urinary copper, serum “free” copper, liver function test/US, slit lamp (for Kayser-Fleischer ring), consider hepatic biopsy with hepatic copper

ADAR1-related BSN	+		++			BSN	Raised CSF neopterins; positive Interferon signature
Paroxysmal sympathetic			++			According with the triggering ABI	HyperCKemia is infrequent
**Metabolic and endocrine disorders**							
Uremic encephalopahty		+ (asterixis)				T2W hyperintensities of BG, thalami, midbrain and mesial temporal lobes.	raised urea and creatinine, hyperkalemia, hyperphosphatemia, hypocalcemia, anemia
Hepatic encephalopahty		+ (asterixis)				T2W hyperintensities of insula, thalami, internal capsule; Possible T1W BG hyperintensities (porto-systemic shunts)	raised aminotransferase levels, coagulopathy, hypoalbuminemia
Hyperglycemia and Diabetes	+					T1W hyperintensity of putamen and/or caudate, high DWI signal	blood glucose levels, EAB, ketones, electrolyte levels (hypernatriemia, hypokaliemia)
Hyperthyroidism and thyrotoxicosis	(+)				++	Usually normal	Thyroid hormones levels (TSH, fT3, fT4), thyroid US, thyroid autoimmunity
Hypercalcemia		+				Depending on the cause (exclude BG dystrophic calcification)	Depending on the underlying disorder
Hypernatremia	+	+			+	Rarely: Transient splenial lesions, Myelinolysis	Depending on the underlying disorder
Hypomagnesemia	+	+			+	Possible cerebellar hyperintensities (usually in chronic forms)	Depending on the underlying disorder

MD phenomenology and relevant neuroimaging and biochemical findings in acute-onset MD in children and adolescents. ++ frequent, + possible, (+) rare
[1]Suggested IEM screening: arterial blood gas, ammonium and lactate levels (blood/CSF), urinary organic acids, plasma acylcarnitines, plasma aminoacids, homocysteine levels, ketones. If suspicion of mitochondrial disorders: Magnetic resonance spectroscopy, pyruvate, thiamine, biotin; consider muscle biopsy for hystology and respiratory chain complexes activity, WB for detection of mtDNA depletion. aAb: autoantibodies; , aPL: antiphospholipid autoantibodies, ASO: Antistreptolysin O; BG: basal ganglia; CPK: creatine phosphokinase; CRP: C-reactive protein, CNS: central nervous system; CSF: cerebrospinal fluid; DWI: diffusion weighted imaging; EKG: electrocardiogram; ESR: erythrocyte sedimentation rate; GABHS: Group A beta-hemolytic streptococcus; GP2: glycoprotein 2, GP: globi pallidi; HVA: homovanillic acid; ICA: internal carotid artery, IEM: inborn errors of metabolism, LAC: Lupus anticoagulant; MRA: magnetic resonance angiography; OCB: oligoclonal bands; T1W: T1 weighted images; T2: T2 weighted, US: ultrasonography, VMA: vanillymandelic acid; WM: white matter.

Illustrative case 1.

A 12 years old girl with no previous relevant medical history presented to the pediatric emergency department for occurrence of generalized involuntary movements, noticed from the previous 2 weeks and gradually worsening. A deterioration of handwriting was noticed in the previous month, associated with an unusual emotional lability. Developmental milestones were unremarkable, and no family history of neurological disorders was present. An episode of pharyngitis two months before was mentioned, but no history of arthralgia was reported. Examination revealed bilateral choreic movements of the face, trunk, upper and lower limbs, associated with mild hypotonia. A positive milkmaid sign (the tendency of patient's finger to squeeze and release when asked to keep a firm grip of examiner's fingers) was present. Involuntary movements were asymmetrical, predominantly affecting the right hemibody. In addition, a systolic heart murmur of 3/6 was detected during pediatric emergency consultation.

Throat swab culture came back positive for GABHS, and a markedly raise antistreptolysin O titer was found. Electrocardiogram was normal, while echocardiogram showed a moderate mitral regurgitation, with thickened valve leaflets – highly suggestive of rheumatic heart disease. Routine blood tests and Brain MRI were normal. A brief cycle of oral prednisone followed by rapid tapering was prescribed. Involuntary movements gradually improved until disappearance of in the following 4 months.

Commentary: SC is the most frequent cause of acute chorea in children. In this case, two major Jones criteria (chorea and rheumatic heart disease) coupled with evidence of previous GABHS infection allow the diagnosis.

Illustrative case 2

A 17 years old boy with mild intellectual disability and behavioral disorders (anxiety, agitation, aggressive behaviors) started a haloperidol treatment - on top of his chronic aripiprazole therapy -because of a recent worsening of his behavioral issues. Few days after beginning the treatment, medical advice was asked for the abrupt appearance of a head tilt. On examination, the patient showed a cervical dystonia combining latero- and retrocollis, with violent dystonic spasms. Haloperidol was suspended, and anticholinergic treatment with trihexyphenidyl was started.

Commentary: ADRs after exposure to potent DRBA are not uncommon in children and adolescents (especially in males), and polytherapy is an adjunctive risk factor.

Illustrative case 3

A 5 years old boy with normal early development presented at the age of 12 months with high fever, dystonic posturing of the neck (torticollis), right arm dystonic movements, marked irritability and cognitive and motor regression with refusal to walk and reduced language output. Symptoms occurred 3 weeks after anti-meningococcal B vaccine.

Routine blood tests showed mildly elevated CRP, and brain MRI demonstrated bilateral striatal T2-hyperintensities and small putamina. MR Spectroscopy did not show a raised lactate peak. Lumbar puncture showed mild elevation of CSF proteins, normal glucose and lactate levels, negative cultures and PCR for neurotropic viruses. Suspecting a defect of energy metabolism, an extensive metabolic panel was performed, including blood ammonium, lactate, acylcarnitines, aminoacids and urinary organic acids, with normal results. Neurotransmitter analysis revealed normal monoamine metabolites, but massively increased neopterins, suggesting an inflammatory process. On this basis, treatment with intravenous immunoglobulin and oral steroids was administered, with partial improvement. However, oligoclonal bands and autoantibody panel for AE came back normal.

Given the young age and the absent evidence for an infectious or autoimmune disorder, a genetic condition was suspected - despite the lack of markers for brain energy defect. A targeted gene panel for pediatric onset-movement disorders revealed the presence of biallelic ADAR1 pathogenic variants and interferon signature confirmed an over-expression of Type I interferon-regulated genes.

Commentary: fever-induced encephalopathy with MDs in toddlers is a challenging scenario. Main differential diagnosis includes CNS infections, autoimmune encephalitis and other autoinflammatory conditions, mitochondrial disorders and IEM (especially in young children). Investigations should prioritize treatable causes. Brain MRI is of great diagnostic value and may orientate further investigations. Nevertheless, some conditions – such as ADAR1-related disorders – presents with confusing features: bilateral and symmetric basal ganglia lesions and young age point towards a genetic-metabolic cause, while inflammatory markers would be in favor of an autoimmune disease. Despite its extreme rarity, ADAR1-related disorders may exemplify the diagnostic process in similar cases.

Illustrative case 4

A 16 years old girl was admitted to the pediatric emergency department for the abrupt appearance of upper limbs tremor during her school lessons, preventing her from taking notes. Waiting for emergency consultation, the upper limbs tremor gradually disappeared, and a shaking tremor of the legs emerged, preventing her to stand and walk. During the visit, she looked distressed and frightened, and was only able to move around in a wheelchair. Legs tremor was present at rest, on standing and in the attempts to walk. It was a high amplitude, predominantly slow tremor, but its frequency showed abrupt transient increases. A positive entrainment of tremor frequency was detected while asking the girl to tap with her right hand at an externally cued rhythm. When her mother was asked to leave the room, the girl said she was experiencing a stressful period after her parents' divorce. A final diagnosis of functional tremor was made.

Commentary: a positive diagnosis of functional MD relies on the demonstration of specific features, including abrupt onset, symptoms variability, distractibility, and incongruence of the phenomenology with an organic disorder.
